# Supplementary figures and images for: A pair of primers facing at the double-strand break site enables to detect NHEJ-mediated indel mutations at a 1-bp resolution
Source: Sci Rep. 2022 Jul 8;12:11681. doi: 10.1038/s41598-022-15776-5 (PMC9270360; doi:10.1038/s41598-022-15776-5)

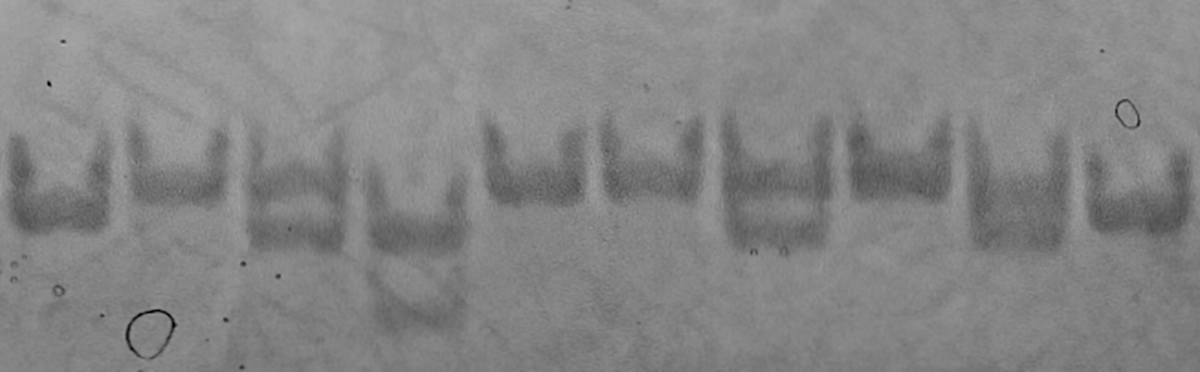

Supplement: Supplementary file 1 — Supplementary Information 1. [file 41598_2022_15776_MOESM1_ESM.tif]

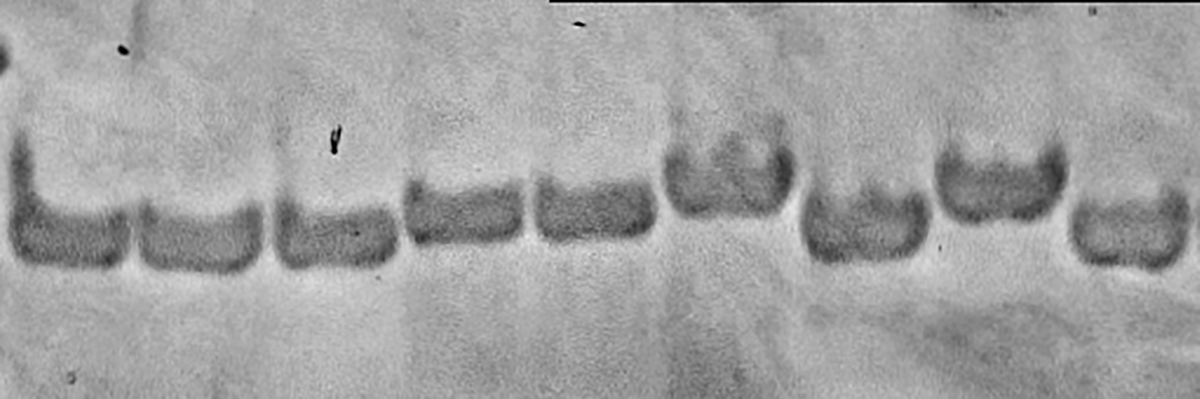

Supplement: Supplementary file 2 — Supplementary Information 2. [file 41598_2022_15776_MOESM2_ESM.tif]

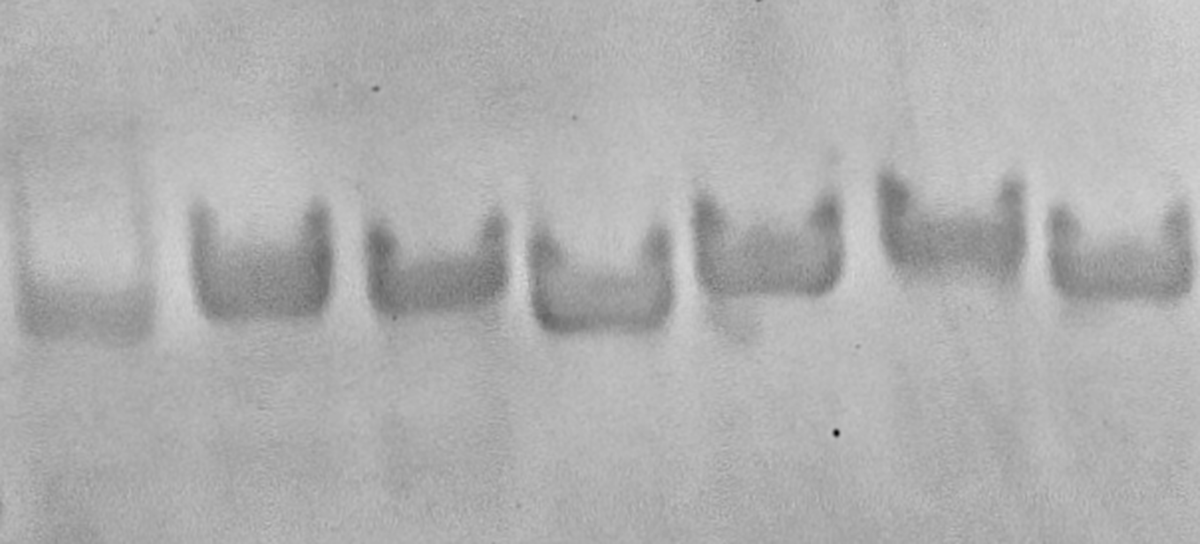

Supplement: Supplementary file 3 — Supplementary Information 3. [file 41598_2022_15776_MOESM3_ESM.tif]

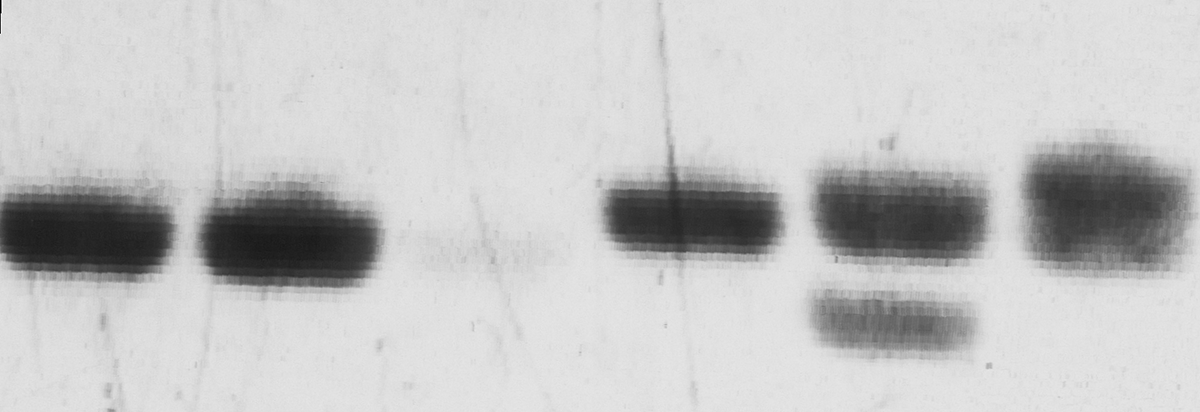

Supplement: Supplementary file 4 — Supplementary Information 4. [file 41598_2022_15776_MOESM4_ESM.tif]

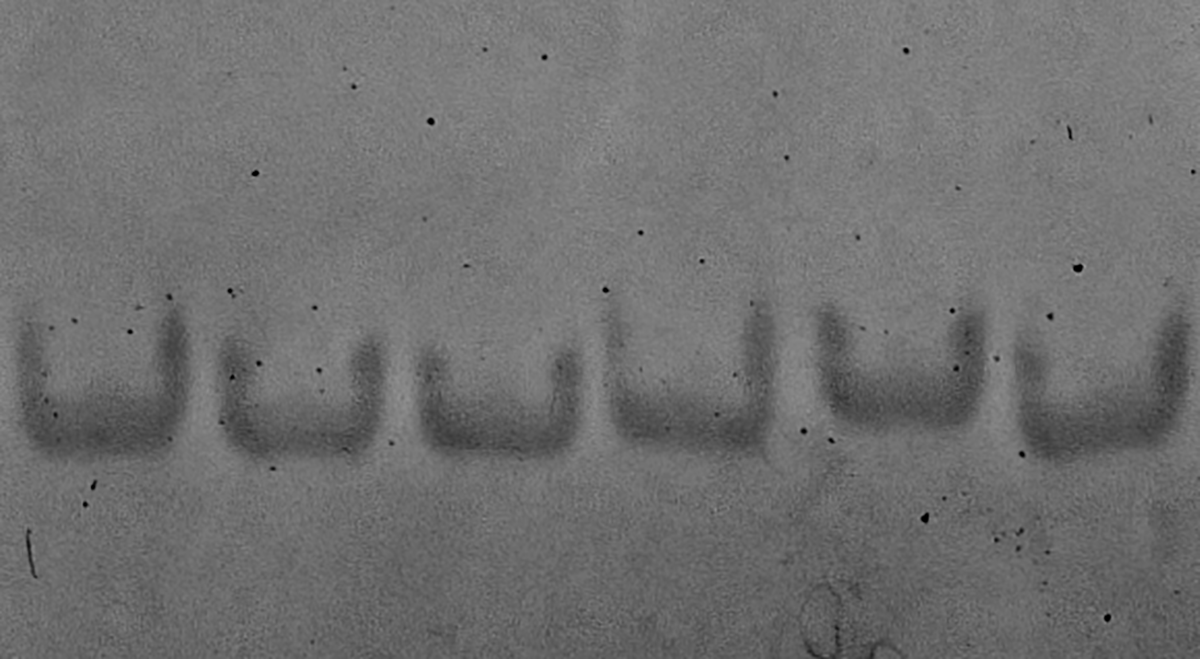

Supplement: Supplementary file 5 — Supplementary Information 5. [file 41598_2022_15776_MOESM5_ESM.tif]

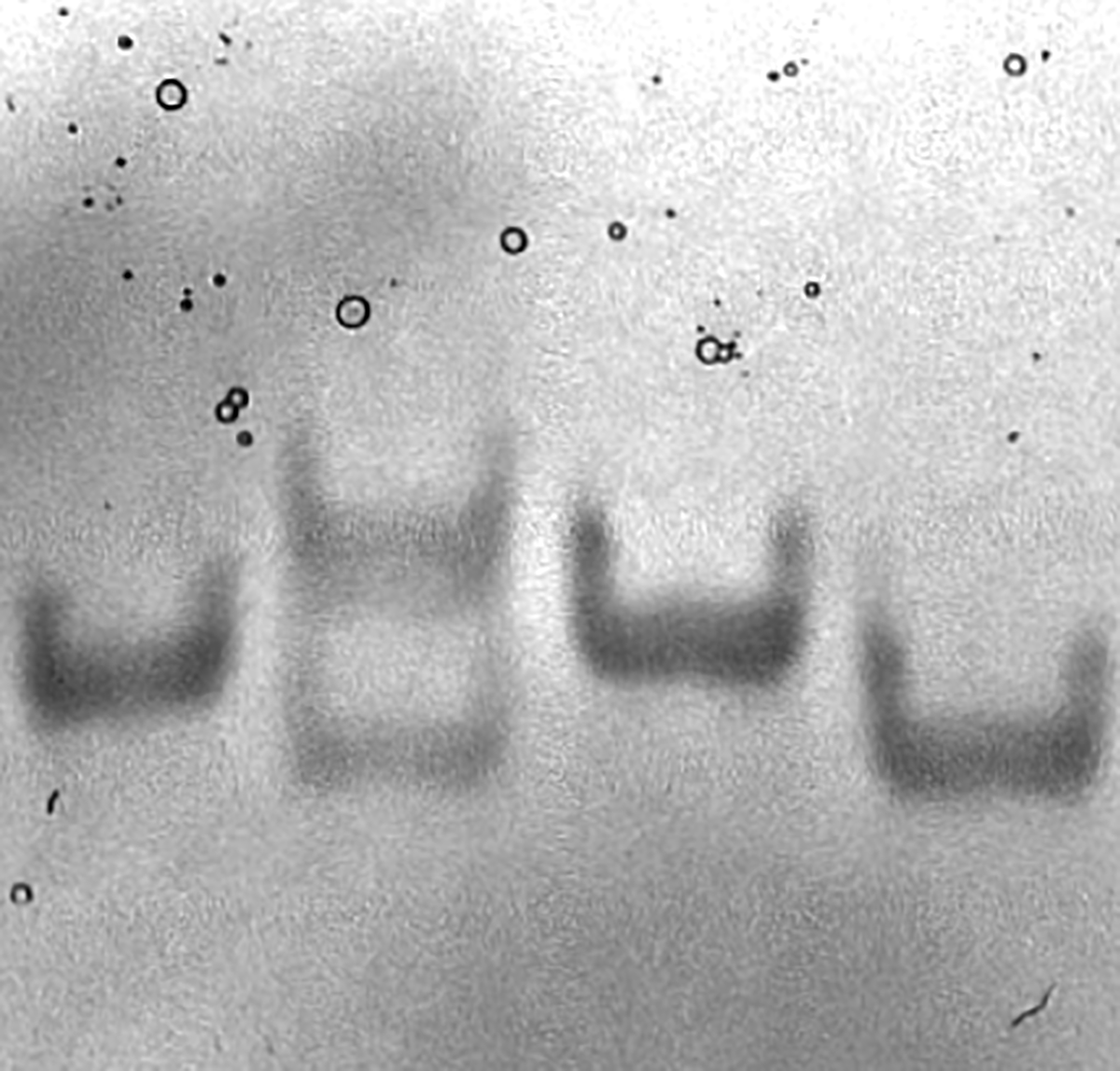

Supplement: Supplementary file 6 — Supplementary Information 6. [file 41598_2022_15776_MOESM6_ESM.tif]
