# Supplementary material for: A pair of primers facing at the double-strand break site enables to detect NHEJ-mediated indel mutations at a 1-bp resolution
Source: Sci Rep. 2022 Jul 8;12:11681. doi: 10.1038/s41598-022-15776-5 (PMC9270360; doi:10.1038/s41598-022-15776-5)

A pair of primers facing at the double-strand break site enables to detect NHEJ-mediated indel mutations at a 1-bp resolution

Faryal Ijaz[1], Ryota Nakazato[1], Mitsutoshi Setou[2], and Koji Ikegami*[1,2,3]

[1]Department of Anatomy and Developmental Biology, Graduate School of Biomedical and Health Sciences, Hiroshima University, 1-2-3 Kasumi, Minami-Ku, Hiroshima 734-8553, Japan

[2]Department of Cellular and Molecular Anatomy and International Mass Imaging Center, Hamamatsu University School of Medicine, Hamamatsu 431-3192, Japan

[3]JST, PRESTO, 4-1-8 Honcho, Kawaguchi, Saitama 332-0012, Japan

Email: k-ikegami@hiroshima-u.ac.jp

Supplementary Table 1. PCR Parameters for DST-PCR screening.

Dync2h1			
**DNA Polymerase**	KOD-Plus-Neo		
	**Temperature**	**Time**	**No.of Cycles**
**Initial Denaturation**	94°C	2 min	1X
**Denaturation**	98°C	10 sec	
**Annealing**	55°C	30 sec	35X
**Extension**	68°C	30 sec	
**Final Extension**	68°C	5 min	1X

Ift144			
**DNA Polymerase**	KOD-Plus-Neo		
	**Temperature**	**Time**	**No.of Cycles**
**Initial Denaturation**	94°C	2 min	1X
**Denaturation**	98°C	10 sec	
**Annealing**	55°C	30 sec	35X
**Extension**	68°C	30 sec	
**Final Extension**	68°C	5 min	1X

Inpp5e			
**DNA Polymerase**	ExTaq		
	**Temperature**	**Time**	**No.of Cycles**
**Initial Denaturation**	94°C	5 min	1X
**Denaturation**	94°C	15 sec	
**Annealing**	65°C	30 sec	35X
**Extension**	72°C	30 sec	
**Final Extension**	72°C	5 min	1X

Arntl			
**DNA Polymerase**	KOD-Plus-Neo		
	**Temperature**	**Time**	**No.of Cycles**
**Initial Denaturation**	94°C	2 min	1X
**Denaturation**	98°C	10 sec	
**Annealing**	60°C	30 sec	35X
**Extension**	68°C	30 sec	
**Final Extension**	68°C	5 min	1X

Pkd1			
**DNA Polymerase**	KOD-Plus-Neo		
	**Temperature**	**Time**	**No.of Cycles**
**Initial Denaturation**	94°C	2 min	1X
**Denaturation**	98°C	10 sec	
**Annealing**	60°C	30 sec	35X
**Extension**	68°C	30 sec	
**Final Extension**	68°C	5 min	1X

Pkd2			
**DNA Polymerase**	KOD-Plus-Neo		
	**Temperature**	**Time**	**No.of Cycles**
**Initial Denaturation**	94°C	2 min	1X
**Denaturation**	98°C	10 sec	
**Annealing**	60°C	30 sec	35X
**Extension**	68°C	30 sec	
**Final Extension**	68°C	5 min	1X

Supplementary Figure Legends

Supplementary Figure 1. Detection of insertion mutations via DST-PCR

(a) TBE-high-resolution-PAGE of DST-PCR products from *Dync2h1-KO* clone#7 and wild-type cells in triplicates showing visibility of 1-bp resolution. Lane Ctrl: PCR product (40-bp) amplified from the genomic DNA of wild-type unedited cells. Clone #7 (* of figure 2**b**) showed a fragment mobility 2-bp upper shift. **(b)** Sanger sequencing of the PCR amplicons from *Dync2h1-KO* candidate clones #3, #4 and #5 (in figure 2**b**) around the PAM sequence. **(c)** TBE-high-resolution-PAGE of DST-PCR products from *Ift-144-KO* clones #4 and wild-type cells in triplicates showing visibility of 1-bp resolution. Lane Ctrl: PCR product (41-bp) amplified from the genomic DNA of wild-type unedited cells. Clone #4 (* of figure 2**f**) showed a fragment mobility 1-bp upper shift **(d)** Sanger sequencing of the PCR amplicons from *Ift-144-KO* candidate clones #2 and #6 (in figure 2**f**) around the PAM sequence. The arrows indicate the locations of the sequencing primers (see Table 1). The 20-bp sgRNA and 3-bp PAM sequences are highlighted in magenta and orange respectively. Wild-type sequence (Ref), base deletion (-), base insertion (▲), base substitution (▲). Original gels are presented in Supplementary Raw Data. Created in Adobe Illustrator CC V26.2.1 (https://adobe.com/products/illustrator).

Supplementary Figure 2. Detection of deletion mutations via DST-PCR

(a) TBE-high-resolution-PAGE of DST-PCR amplicons from *Inpp5e-KO* clone#4 and wild-type cells in triplicates showing visibility of 1-bp resolution. Lane Ctrl: PCR product (39-bp) amplified from the genomic DNA of wild-type unedited cells. Clone #4 (* of figure 3b) showed a fragment mobility 1-bp upper and lower shift. **(b)** Sanger sequencing of the PCR amplicons from *Arntl-KO* candidate clones #1 and #7 (in figure 3**e**) around the PAM sequence. The arrows indicate the locations of the sequencing primers (see Table 1). The 20-bp sgRNA and 3-bp PAM sequences are highlighted in magenta and orange respectively. Wild-type sequence (Ref), base deletion (-), base insertion (▲), base substitution (▲). **(c)** TBE-high-resolution-PAGE of DST-PCR products from *Arntl-KO* clone#3 and wild-type cells in triplicates showing visibility of 1-bp resolution. Lane Ctrl: PCR product (41-bp) amplified from the genomic DNA of wild-type unedited cells. Clone #3 (* of figure 3e)

showed a fragment mobility 1-bp and 4-bp lower shift. Original gels are presented in Supplementary Raw Data. Created in Adobe Illustrator CC V26.2.1 (https://adobe.com/products/illustrator).

Supplementary Figure 3. Detection of long deletion mutations via DST-PCR

(a) Scheme of the Pkd1 guide RNA targeting exon 15 in the murine *Pkd1* gene. **(b)** DST-PCR for screening mutations. Upper panel: The arrows indicate the locations of the DST-PCR primers (see Table 1). Bottom panel: TBE-high-resolution-PAGE of DST-PCR products. Lane Ctrl: PCR product (41-bp) amplified from the genomic DNA of wild-type unedited cells. Clones #2 and #3 (*) showed no band amplification (red arrowhead). **(c)** Sanger sequencing of the PCR products of clones #2 and #3 (* in panel **b**) around the PAM sequence (orange rectangle). The arrows indicate the locations of the sequencing primers (see Table 1). **(c)** Sanger sequencing of the PCR products of clones #2 and #3 (* in panel **b**) around the PAM sequence (orange rectangle). Split sequences were shown under the spectrum. Start of split sequences are highlighted as black rectangle. The arrows indicate the locations of the sequencing primers (see Table 1). The 20-bp sgRNA and 3-bp PAM sequences are highlighted in magenta and orange respectively. Wild-type sequence (Ref), base deletion (-).Original gels are presented in Supplementary Raw Data. Created in Adobe Illustrator CC V26.2.1 (https://adobe.com/products/illustrator).

Supplementary Figure 4.

(a) Scheme of the Pkd1 guide RNA targeting exon 11 in the murine *Pkd1* gene. **(b)** DST-PCR for screening mutations. Upper panel: The arrows indicate the locations of the DST-PCR primers (see Table 1). Bottom panel: TBE-high-resolution-PAGE of DST-PCR products. Lane Ctrl: PCR product (39-bp) amplified from the genomic DNA of wild-type unedited cells. Clone #9 (*) showed a fragment mobility upper shift (red arrowhead). **(c)** Sanger sequencing of the PCR products of clone #9 (* in panel **b**) around the PAM sequence (orange rectangle). The arrows indicate the locations of the sequencing primers (see Table 1). The 20-bp sgRNA and 3-bp PAM sequences are highlighted in magenta and orange respectively. Wild-type sequence (Ref), base insertion (▲). **(d)** TBE-high-resolution-PAGE of DST-PCR products in triplicates showing visibility of 1-bp resolution. Lane Ctrl: PCR product (39-bp) amplified from the genomic DNA of wild-type unedited cells. Clone #9 (* in

panel **b**) showed a fragment mobility 1-bp upper shift. Original gels are presented in Supplementary Raw Data. Created in Adobe Illustrator CC V26.2.1 (https://adobe.com/products/illustrator).

Supplementary Figure 1

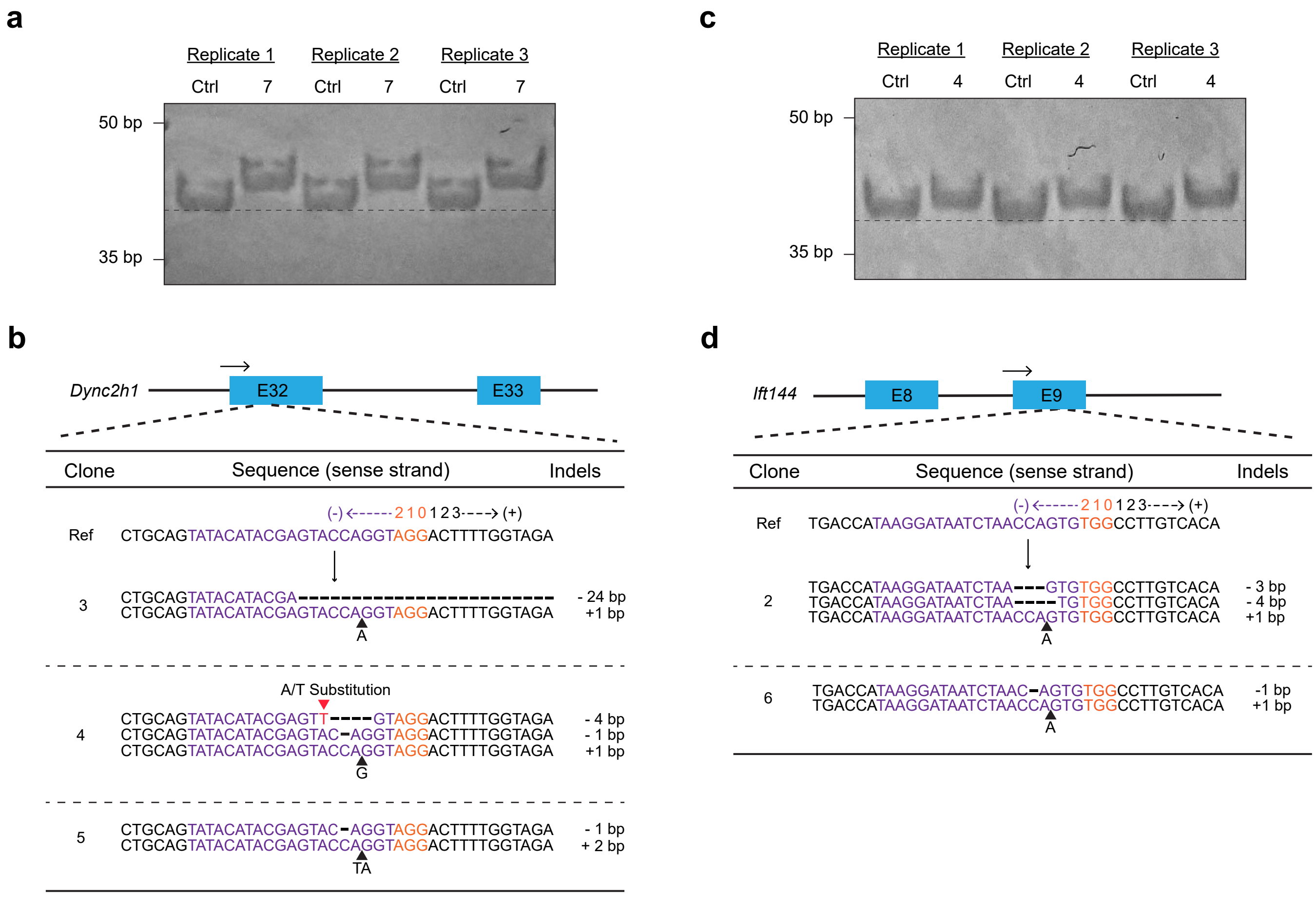

Supplementary Figure 2

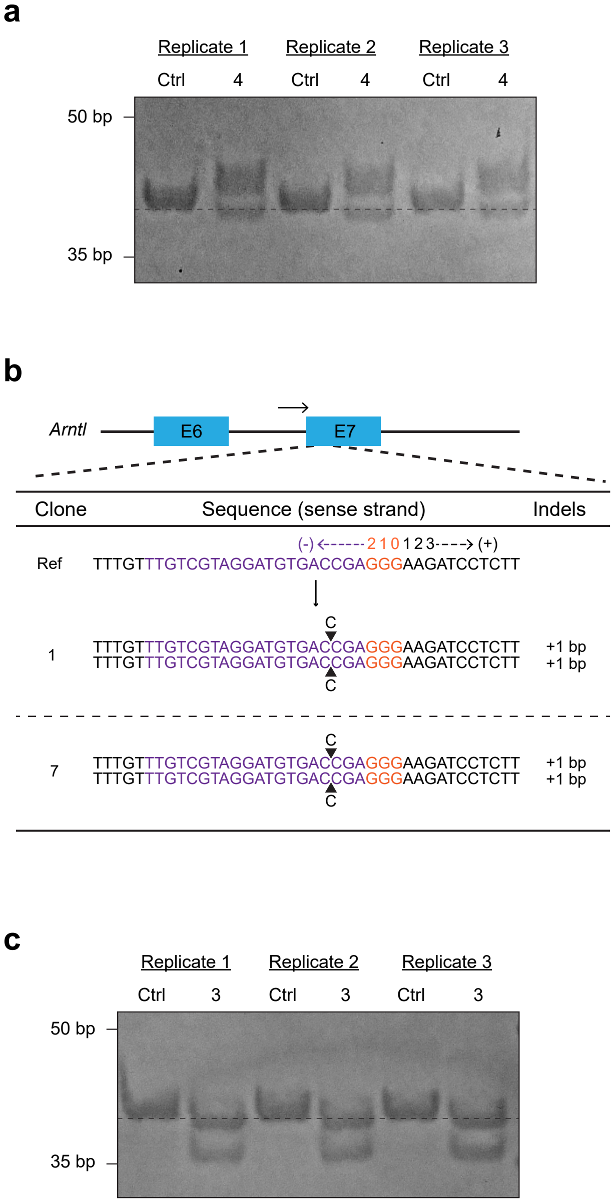

Supplementary Figure 3

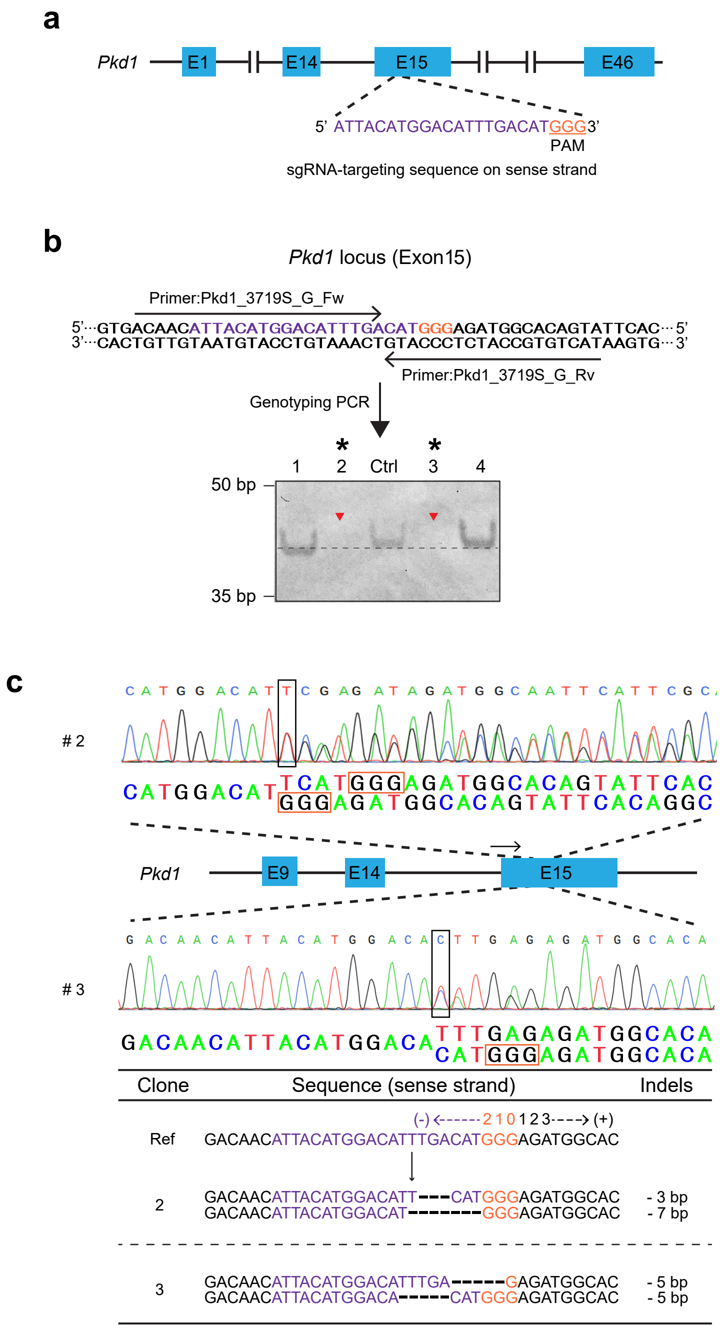

Supplementary Figure 4

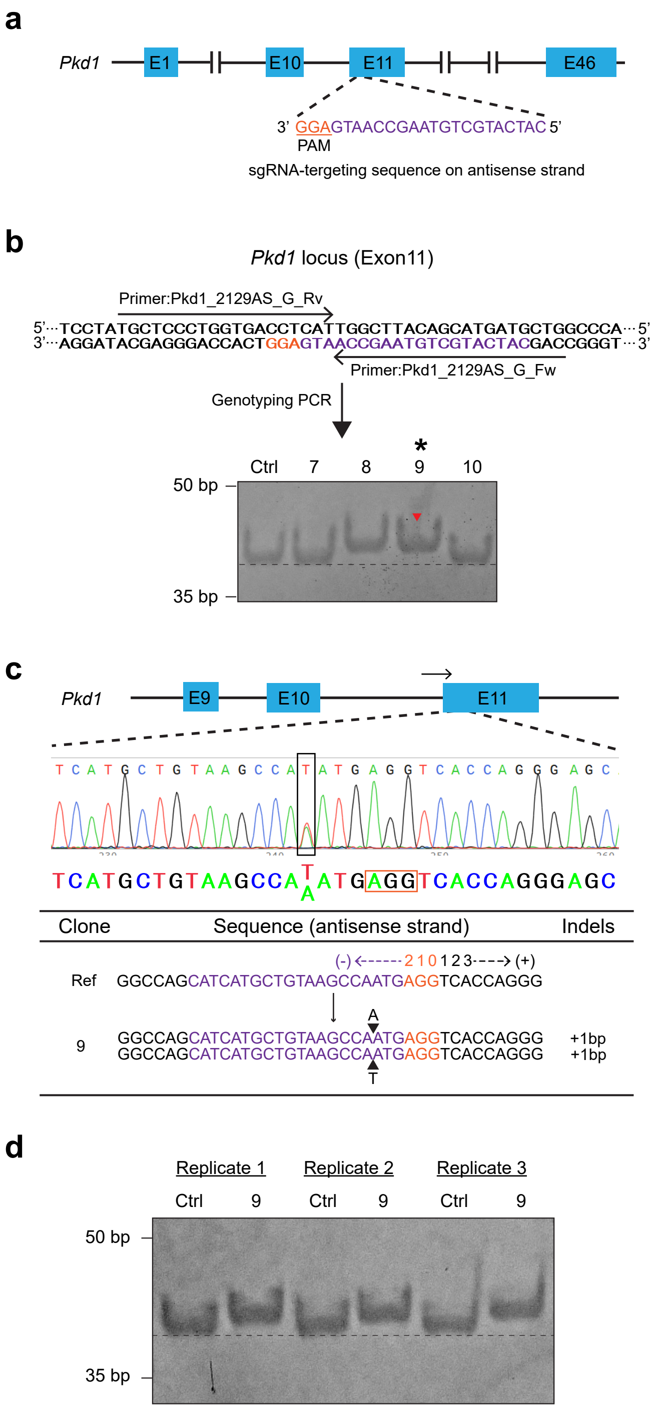

Supplement: Supplementary file 7 — Supplementary Information 7. [file 41598_2022_15776_MOESM7_ESM.pdf]
