# Supplementary material for: A pair of primers facing at the double-strand break site enables to detect NHEJ-mediated indel mutations at a 1-bp resolution
Source: Sci Rep. 2022 Jul 8;12:11681. doi: 10.1038/s41598-022-15776-5 (PMC9270360; doi:10.1038/s41598-022-15776-5)

Raw Data for Figures in Main Text

Dync2h1

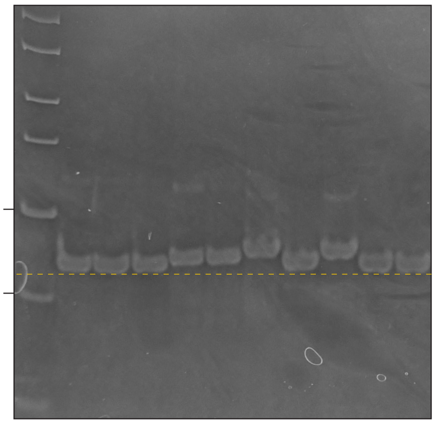

Ift-144

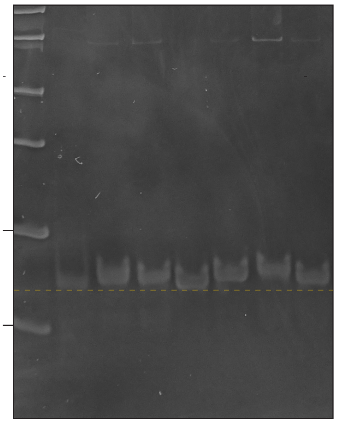

Inpp5e

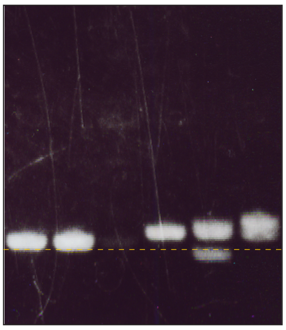

Arntl

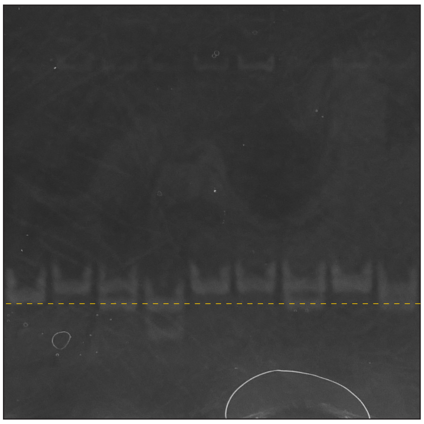

Pkd1

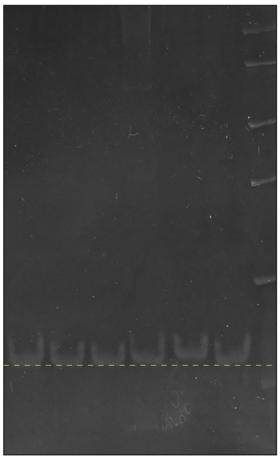

Pkd2

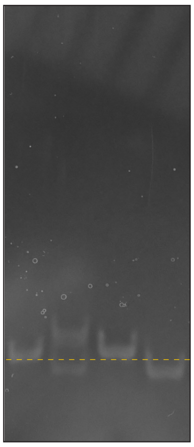

Raw Data for Supplementary Figure 1

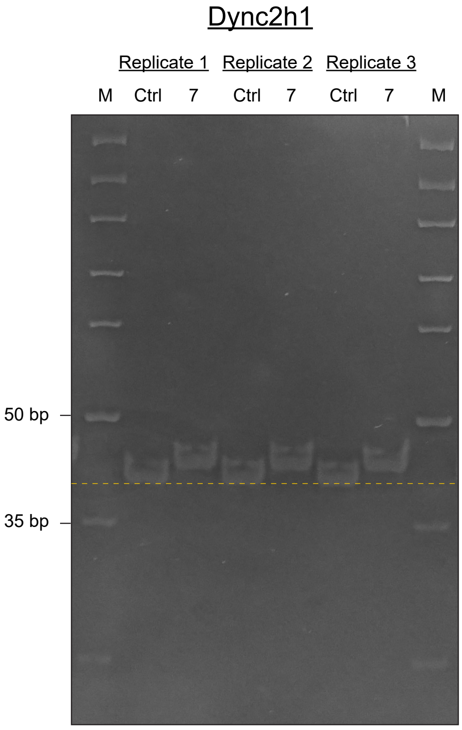

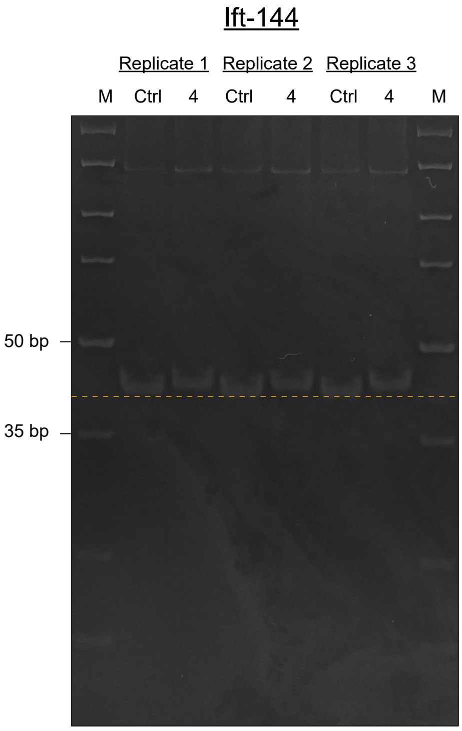

Raw Data for Supplementary Figure 2

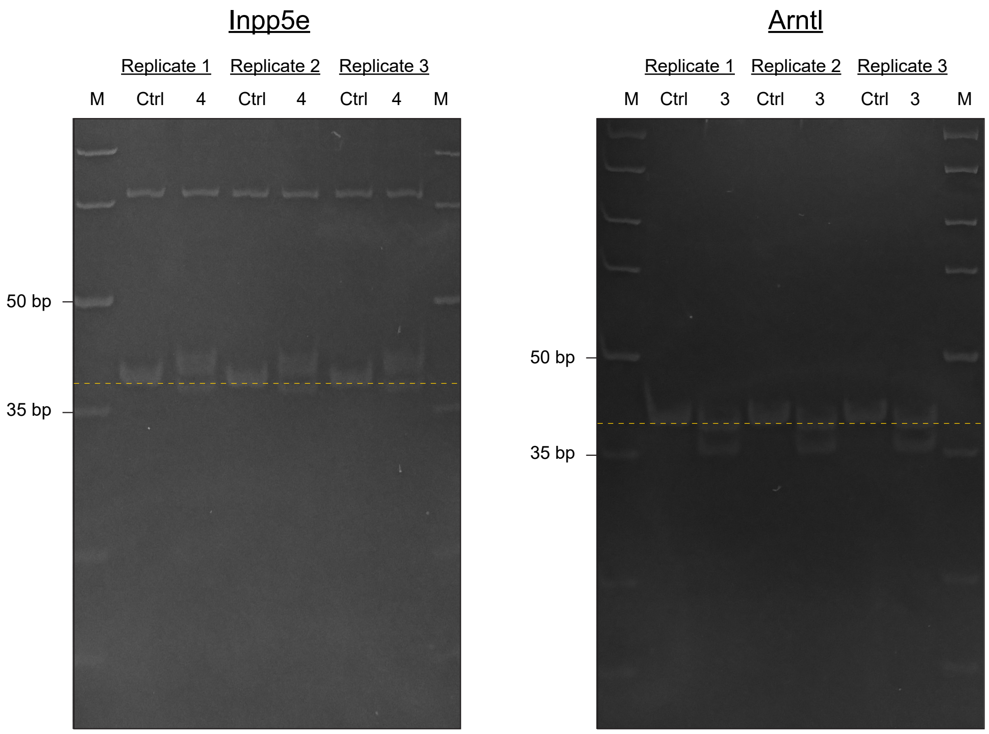

Raw Data for Supplementary Figure 3

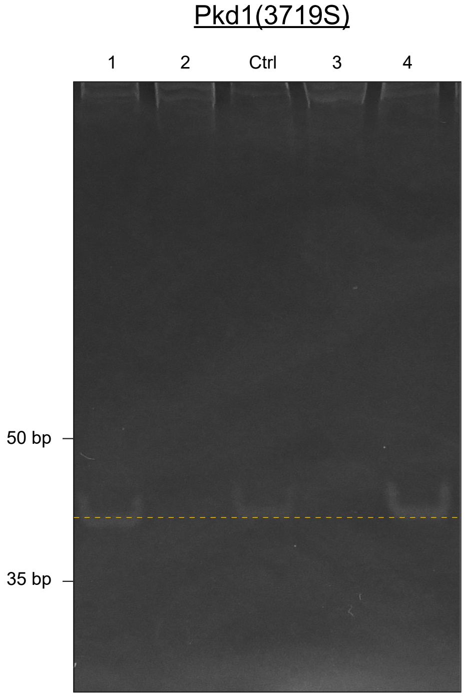

Raw Data for Supplementary Figure 4

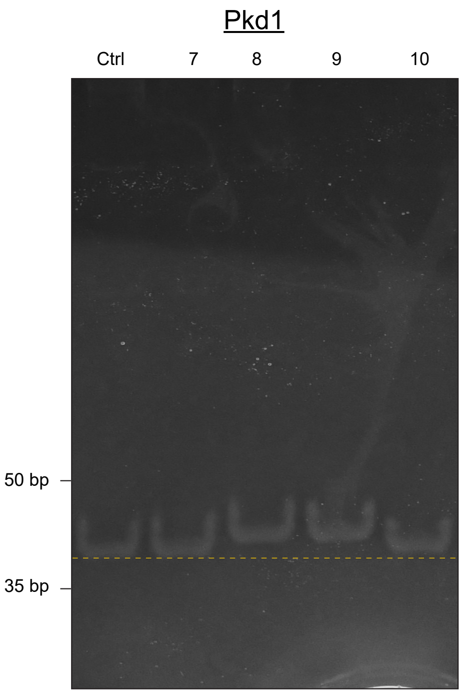

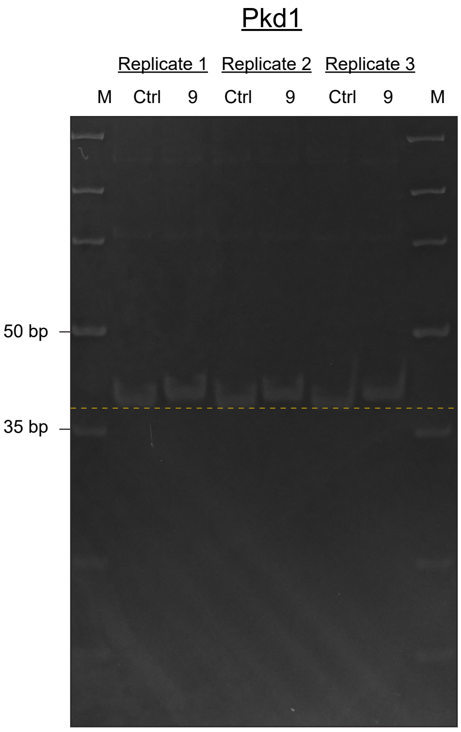

Supplement: Supplementary file 8 — Supplementary Information 8. [file 41598_2022_15776_MOESM8_ESM.pdf]
